# Cyclical mastalgia as a marker of breast cancer susceptibility: results of a case-control study among French women.

**DOI:** 10.1038/bjc.1992.198

**Published:** 1992-06

**Authors:** G. Plu-Bureau, J. C. Thalabard, R. Sitruk-Ware, B. Asselain, P. Mauvais-Jarvis

**Affiliations:** Department of Reproductive Endocrinology, Hôpital Necker, Paris, France.

## Abstract

A matched case-control study in a population of urban, non-menopaused women living in Paris was performed between 1983 and 1985 to investigate the risk of breast cancer (BC) in relation to various factors with a particular interest in the effect of the use of oral contraceptive (OC) and the existence of cyclical mastalgia (CM). Two hundred and ten non-menopaused women, less than 45 years old, with newly diagnosed BC were compared to 210 controls from the same geographic area matched on year of birth, age, education level and age at first full term pregnancy (FFTP), when justified. The adjusted Relative Risk of BC (RRa) was significantly increased for a total duration of OC use longer than 72 months (RRa 2.80; 95% CI 1.56-5.01), as well as the RRa for OC use above 48 months before FFTP (3.26 95% CI 1.37-7.76) and, to a lesser extent, the RRa for OC use above 48 months after FFTP (2.02 95% CI 1.07-3.84) respectively. Adjustment was performed on familial history of BC, personal history of Benign Breast Disease (BBD), age at menarche. A previous history of cyclical mastalgia was found to be associated with an increased risk of BC. The significant increase remained after adjustment on the previously mentioned confounding factors and OC use: RRa 2.12; 95% CI (1.31-3.43). Under a precise definition related to the hormonal environment, mastalgia appear to be an interesting marker of breast cell susceptibility, the importance of which can only be validated by prospective studies.


Br. J. Cancer (1992), 65, 945-949 © Macmillan Press Ltd., 1992

### Cyclical mastalgia as a marker of breast cancer susceptibility: results of a case-control study among French women

G. Plu-Bureau[1,2], J.C. Thalabard[1,2,*], R. Sitruk-Ware[1,**], B. Asselain[3] & P. Mauvais-Jarvis[1]

[1]*Department of Reproductive Endocrinology, 75015 Hôpital Necker, Paris;* [2]*Unité INSERM U292, Epidemiology of Human Reproduction, Hôpital de Bicêtre, 94227 Le Kremlin-Bicêtre Cdx;* [3]*Department of Biostatistics, Institut Curie, 75005 Paris, France.*

**Summary** A matched case-control study in a population of urban, non-menopaused women living in Paris was performed between 1983 and 1985 to investigate the risk of breast cancer (BC) in relation to various factors with a particular interest in the effect of the use of oral contraceptive (OC) and the existence of cyclical mastalgia (CM). Two hundred and ten non-menopaused women, less than 45 years old, with newly diagnosed BC were compared to 210 controls from the same geographic area matched on year of birth, age, education level and age at first full term pregnancy (FFTP), when justified.

The adjusted Relative Risk of BC ($RR_a$) was significantly increased for a total duration of OC use longer than 72 months ($RR_a$ 2.80; 95% CI 1.56-5.01), as well as the $RR_a$ for OC use above 48 months before FFTP (3.26 95% CI 1.37-7.76) and, to a lesser extent, the $RR_a$ for OC use above 48 months after FFTP (2.02 95% CI 1.07-3.84) respectively. Adjustment was performed on familial history of BC, personal history of Benign Breast Disease (BBD), age at menarche.

A previous history of cyclical mastalgia was found to be associated with an increased risk of BC. The significant increase remained after adjustment on the previously mentioned confounding factors and OC use: $RR_a$ 2.12; 95% CI (1.31-3.43).

Under a precise definition related to the hormonal environment, mastalgia appear to be an interesting marker of breast cell susceptibility, the importance of which can only be validated by prospective studies.

Mastalgia or mastodynia are a frequent complaint in general practice (Preece *et al.*, 1976; 1978; Fentiman *et al.*, 1986). Their cumulated incidence rate in women has been estimated by some authors (Kallner, 1985; Maddox *et al.*, 1989) to be as high as 45% during the genital life. They are sometimes considered as part of fibrocystic disease (Sitruk-Ware *et al.*, 1977; Vorherr *et al.*, 1986), although their evidence and somatic relevance are frequently denied (Love *et al.*, 1982; Dupont & Page, 1985).

Several characteristics of mastalgia seem to establish a relation between their occurrence and breast oestrogen impact, namely its bilaterality and periodicity in relation with the menstrual phases, its disappearance after surgical or chemical castration or after anti-oestrogen therapy like progestins or tamoxifen, its possible exacerbation or occurrence under oestrogen or oral contraceptive use.

Hormonal risk factors for breast cancers have been extensively studied in the past 20 years (MacMahon *et al.*, 1973; Kelsey, 1979; Korenman, 1980; Cowan *et al.*, 1981; Henderson *et al.*, 1982; Kelsey & Berkowitz, 1988; Krieger, 1989). Several authors (MacPherson *et al.*, 1987*a*; Muir, 1990) have already pointed out the long delay between the emergence of the first cancerous clone and the breast cancer diagnosis and much effort has been put on the characterisation of high risk populations, and on the delineation of more susceptible periods like the period starting at menarche and ending at first full term pregnancy (FFTP).

More recently, Leinster *et al.* (1987) showed a higher incidence of high risk mammographic patterns in women with cyclical mastalgia and speculated about a possible link with later occurrence of breast cancer.

To investigate the role of cyclical mastalgia as a potential precocious marker of breast steroid susceptibility in relation to breast cancer, we performed a case control study of 210 newly diagnosed breast cancers and 210 age-, year of birth-, age at FFTP, if any, and socioeconomic status-matched controls.

### Materials and methods

### *Case and control selection*

All women with primary diagnoses of breast cancer between 1 January 1983 and 31 December 1985 in Institut Curie, Paris, were included into the study provided they were white caucasian, non-menopaused and their breast cancer diagnosis occurred before their 45th birthday, for the sake of comparability with other studies (Pike *et al.*, 1983; Cancer and Steroid Hormone Study (CASH), 1986; MacPherson *et al.*, 1987*b*; Miller *et al.*, 1989; Olsson *et al.*, 1989; Stadel *et al.*, 1989; UK National Case-Control Study Group, 1989; Clavel *et al.*, 1991). Institut Curie is one of the major cancer units in Paris. The date of diagnosis was the date of the first positive biopsy.

For every case, a control was randomly selected among white caucasian female patients living in the same geographic area and enrolled on a voluntary basis in a chronic health care program. This program consisted in a yearly medical examination including breast examination. To be eligible, a control should have a normal breast examination at the time of interview and no past or present metabolic contra-indication to oral contraceptive use or oestrogen therapy, like glycaemic or lipidic or clotting factor abnormalities.

The probability of exposure to exogenous sex hormones either as oral contraceptive or as medical treatment for menstrual disorders is clearly age-dependent. Furthermore the available exogenous sexual steroids have changed over the past 30 years. Finally educational level and age at first full time pregnancy have been shown to be significant risk factors for breast cancer. Therefore, controls were matched to cases simultaneously by age, year of birth, educational level and age at FFTP when appropriate. The absolute difference between the ages of a pair of matched case and control was equal or less to 2 years. The same limit was adopted as far as

Correspondence: G. Plu-Bureau, Department of Reproductive Endocrinology, Hopital Necker, 149, Rue de Sèvres, 75015 Paris, France.
*Present addresses: Clinical Pharmacology Department, 162 Ave Lacassagne, 69424 Lyon Cdx 03, France; **CIBA-Geigy Ltd., Medical Department, PO Box 4002, Basel, Switzerland.
Received 3 September 1991; and in revised form 10 February 1992.

age at FFTP was concerned. Educational level was coded according to the INSEE (National Institute for Statistical and Economical Studies) classification, i.e. into six categories (1) below or equal to the 6th grade; (2) Middle School; (3) 9th grade; (4) High School completed; (5) College level; (6) University level.

All the cases and the controls gave their informed consent to fill in the questionnaire.

Between 1 January 1983 and 31 December 1985, 573 new breast cancers were detected in Institut Curie in non menopaused women. Among these patients, 210 were less than 45 years old. During the same period of time, 703 premenopaused women were recruited into the Chronic Health Care program and among them, 408 were less than 45 years old. This control population was sorted according to the BC cases, i.e. each BC case was associated with a sub-group of controls matched on age within 2 years, educational level and age at FFTP within 2 years when appropriate. For each case, a control was randomly selected inside the corresponding group thus ensuring a 1:1 matching.

### *Interview*

Each person enrolled into the study was asked a specific standardised structured questionnaire by an interviewer.

The two interviewers were female medical doctors qualified in breast pathology. Before the beginning of the study, all the interviewers were trained for 2 months in managing their interview and in completing the questionnaire always in the same manner. The questionnaire had been previously pretested for 1 year by a team composed of one senior clinician (R.S.W.) and one senior epidemiologist (J.F.) in the breast clinic of the Department of Reproductive Endocrinology until we reached an unambiguous questionnaire.

The interview lasted about 20 min and included basic demographic informations (age, educational level, occupation, marital status, height and weight), reproductive history (age at menarche; menstrual experience; pregnancies; medical history including benign breast disease history), detailed oral contraceptives use namely before and after FFTP if any, familial history of breast cancer, history of previous premenstrual mastalgia.

Oral contraceptive uses were elicited by constructing a monthly calendar of events starting at menarche up to the time of the interview. Contraceptive use was associated with marking events like first sexual intercourse, marriage, FFTP. Information on brand names used was elicited with the aid of good colour prints of the packaging, showing its front and back and its content. Oral contraceptives used by the patients of this study were recorded according to the three following categories; (1) standard dose oral contraceptives (SDC) containing 50 micrograms (μg) per pill of ethynil-estradiol (EE); (2) Low oral dose contraceptives (LDC) containing less than 40 μg per pill of EE; (3) continuous microprogestative pill (P). However, the limited number of patients exposed to the two last categories did not allow us to take into account this variable and only global results are presented.

### *Definition of cyclical mastalgia*

As the concept of mastalgia is rather controversial, we adopted very stringent criteria and interviewers were more particularly trained in this regard. Details of the questions can be found in appendix. Cyclical mastalgia (CM) was defined as a bilateral painful breast swelling, lasting for more than 4 days and up to 3 weeks, always preceding menses and subsiding during menstruation. This monthly event was noted only if it persisted for more than 6 months. This definition clearly discarded non cyclical mastalgia and Tietze's disease and is in accordance with classification proposed by previous authors (Preece et al., 1976; Wisbey et al., 1983). This symptom was recorded separately for various phases of genital life, i.e. during the period starting at menarche until FFTP, and after FFTP if the interviewee had already been pregnant, otherwise until the time of the interview. For each type of OC used were recorded the age at first use, the total duration of use, and, when it was relevant, the existence, exact chronology and duration of breast pain and swelling.

### *Statistical analysis*

Comparisons between groups were performed using the student's $t$-test for paired and unpaired quantitative data and chi square test for qualitative data.

We used the multivariate logistic regression methods for matched case-control studies (Breslow & Day, 1978).

The strategy of performing a 1:1 analysis exposed us to overmatching as about 20% of cases could be associated with the same control. Following the recommendations of several authors (Breslow & Day, 1980; Brookmeyer et al., 1986), we initially performed a general matched analysis with unequal strata and unequal cases and controls within each stratum using a modified version of the Breslow and Day program (Thalabard et al., 1989). Then we compared the result of this global analysis with the results of multiple matched analysis obtained by randomly picking out, for each case, a control from the group of possible matched control subjects (B. Falissard, data unpublished). As we did not observe striking differences between the results from the different analysis on all studied variables, either before or after adjustment, only results from a matched 1:1 analysis are shown.

Tables with a summary of the respective counts for cases and controls in different situation are shown, but should be interpreted with caution, as they do not take specifically into account the matched pairs contrary to the calculations of the RR. Unadjusted and adjusted relative risk according to classical potential confounding variables (familial history of breast cancer, age at menarche, previous benign breast disease) with their corresponding 95% confidence interval (95% CI) were computed.

### Results

A summary of cases and controls is shown in Table I. No significant differences were found as far as age at menarche, age at first OC use are concerned. The total durations of OC use were statistically significant, while the durations of OC use before or after FFTP did not differ between the two groups, but corresponded to smaller sample sizes.

The influence of previously published BC risk factors like familial history of breast cancer, personal history of BBD, age at menarche is considered in Table II. The observed adjusted RR for familial history of BC and personal history of BBD are 2.89 (1.53–5.43) and 5.55 (2.60–11.87) respec-

**Table I** Summary of cases and controls results expressed as mean (standard deviation)

| | *Cases mean (s.d.)* | *Controls mean (s.d.)* | *Significance P* |
|---|---|---|---|
| Age (years) | 36.9 (4.5) ($n$ = 210) | 36.8 (4.5) ($n$ = 210) | N.S. |
| Menarche (years) | 12.7 (1.5) ($n$ = 210) | 12.8 (1.5) ($n$ = 210) | N.S. |
| Age at FFTP | 24.7 (4.2) ($n$ = 173) | 24.9 (4.5) ($n$ = 173) | N.S. |
| Age at first OC use before FFTP | 22.8 (3.9) ($n$ = 72) | 23.4 (4.2) ($n$ = 63) | N.S. |
| Duration of OC use before FFTP (months) | 19.7 (40.4) ($n$ = 72) | 13.2 (29.3) ($n$ = 63) | N.S. |
| Duration of OC use after FFTP (months) | 33.3 (45.7) ($n$ = 125) | 25.5 (41.2) ($n$ = 109) | N.S. |
| Total duration of OC use (months) | 52.9 (53.1) ($n$ = 162) | 38.8 (47.6) ($n$ = 143) | 0.004 |

The number $n$ in brackets indicates the number of subjects exposed to the factor. Last column: $P$-value (two-tailed situation). N.S. corresponds to $P > 0.05$.

**Table II** Analysis of data according to classical risk factors

| | *Cases* *n(%)* | *Controls* *n (%)* | *Unadjusted RR* *(95% CI)* | *Adjusted RR*[a] *(95% CI)* |
|---|---|---|---|---|
| Age at menarche (yr) | | | | |
| <12 | 44 (21) | 41 (20) | 1.10 (0.63–1.91) | 1.43 (0.80–2.58) |
| [12–13] | 46 (22) | 53 (25) | 0.89 (0.52–1.51) | 0.98 (0.56–1.73) |
| [13–14] | 55 (26) | 50 (24) | 1.13 (0.62–1.90) | 1.08 (0.62–1.90) |
| [14- | 65 (31) | 66 (31) | 1.00 | 1.00 |
| Familial history of BC | 54 (26) | 22 (11) | 3.0 (1.68–5.35) | 2.89 (1.53–5.43) |
| Personal history of BBD | | | | |
| No | 159 (76) | 198 (94) | 1.00 | 1.00 |
| Yes | 51 (24) | 12 (6) | 5.33 (2.58–11.03) | 5.55 (2.60–11.87) |
| Nodular hyperplasia | 15[b] (7) | 5 (3) | 4.36 (1.28–14.78) | 4.53 (1.32–15.55) |
| Fibrocystic disease | 32[b] (15) | 4 (2) | 8.74 (3.01–25.37) | 9.11 (2.90–28.62) |
| Others | 6[b] (3) | 3 (1) | 2.00 (0.50–8.00) | 1.59 (0.35–7.17) |
| Oral contraceptive use | | | | |
| Never | 48 (23) | 67 (32) | 1.00 | 1.00 |
| 1–24 months | 41 (20) | 53 (25) | 1.08 (0.62–1.88) | 1.23 (0.68–2.23) |
| 25–72 months | 57 (27) | 49 (23) | 1.62 (0.95–2.77) | 2.00 (1.13–3.55) |
| >72 months | 64 (30) | 41 (20) | 2.18 (1.27–3.74) | 2.80 (1.56–5.01) [<0.01)[c] |
| Oral contraceptive use before FFTP | | | | |
| Never | 138 (66) | 147 (70) | 1.00 | 1.00 |
| 1–24 months | 15 (7) | 19 (9) | 0.96 (0.44–2.08) | 1.62 (0.65–4.06) |
| 25–48 months | 26 (12) | 26 (12) | 1.08 (0.54–2.14) | 1.44 (0.69–3.23) |
| >48 months | 31 (15) | 18 (9) | 2.19 (1.03–4.69) | 3.26 (1.37–7.76) [<0.05][c] |
| Oral contraceptive use after FFTP | | | | |
| Never | 85 (40) | 101 (48) | 1.00 | 1.00 |
| 1–24 months | 48 (23) | 50 (24) | 1.34 (0.77–2.35) | 1.42 (0.75–2.68) |
| 25–48 months | 17 (8) | 16 (8) | 1.26 (0.58–2.73) | 1.64 (0.66–4.09) |
| >48 months | 60 (29) | 43 (20) | 1.92 (1.09–3.37) | 2.02 (1.07–3.84) [<0.05][c] |

[a]Covariates of the adjusted model: familial history of BC, personal history of BBD, age at menarche, [3]oral contraceptive use. [b]Counts do not sum up to 51 as some women could have had more than one type of BBD. [c]*P*-value for significant trend.

tively and thus statistically different from one at the 5% risk. The adjusted RR associated with total duration of OC use reached a significant level when used more than 6 years (72 months). The RRa corresponding to a total duration of OC use longer than 4 years, respectively before and after FFTP, reached statistical significance as well. Data related to the various subclasses of BBD indicate a higher rate of nodular hyperplasia and fibrocystic disease in the case-group than in the control-group, thus leading to increased RRa of BC in relation to these two types of BBD.

Table III summarises data related to cyclical mastalgia. CM was associated with a 2-fold increase of RR (2.12; 95% CI 1.31–3.43), with a symptom-duration effect ($P<0.0001$). In all three situations considered, i.e. before FFTP, after FFTP or during OC use, the adjusted RR of BC associated with mastalgia was significantly higher than unity, ranging from 2.09 (95% CI 1.04–4.19) to 2.24 (95% CI 1.19–4.24).

The adjusted relative risk of cyclical mastalgia on BC in the various subgroups of BBD was respectively 2.06 (95% CI 1.28–3.34) in the patients with no previous history of BBD as compared with 8.27 (95% CI 1.12–61.21) with a previous history of nodular hyperplasia and/or fibroadenomatosis, 6.52 (95% CI 0.96–44.40) with a previous history of fibrocystic disease and 3.33 (95% CI 0.20–55.87) with other BBD. The limited number of pairs contributing to the determination of the coefficients in the underlying statistical model

**Table III** Summary of the data corresponding to the symptom cylical mastalgia (CM). The denominator used in the calculation of the % is 210 with exceptions indicated in brackets

| | *Cases* *n(%)* | *Controls* *n (%)* | *Unadjusted RR* *(95% CI)* | *Adjusted RR*[a] *(95% CI)* |
|---|---|---|---|---|
| Cumulated duration of CM (months) | | | | |
| <6 | 113 (54) | 161 (77) | 1.00 | 1.00 |
| 6–48 | 36 (17) | 32 (15) | 1.54 (0.90–2.62) | 1.12 (0.61–2.05) |
| 49–96 | 16 (8) | 6 (3) | 3.41 (1.28–9.08) | 2.24 (0.77–6.52) |
| 97– | 45 (21) | 11 (5) | 6.46 (2.87–14.53) [0.0001][b] | 5.54 (2.79–13.39) [<0.0001][b] |
| Total CM | 97 (46) | 49 (23) | 2.66 (1.72–4.11) | 2.12 (1.31–3.43) |
| CM before FFTP | 51 (24) | 23 (11) | 2.65 (1.50–4.68) | 2.24 (1.19–4.24) |
| CM after FFTP | 74 (43) (*n* = 173) | 35 (20) (*n* = 173) | 2.77 (1.69–4.56) | 2.19 (1.26–3.81) |
| CM while under OC use | 34 (21) (*n* = 162) | 16 (11) (*n* = 143) | 2.31 (1.15–4.74) | 2.09 (1.04–4.19) |

[a]Covariates of the adjusted model: familial history of breast cancer, personal history of benign breast disease (BBD), age at menarche, oral contraceptive use. [b]*P*- value for significant trend.

explains the large CI. In order to limit the number of variables incorporated into the model, adjustment was performed here only on the BBD type-coding variables, cyclical mastalgia and the corresponding interactions. Therefore, calculated numbers cannot be directly compared to the other adjusted RR shown in Table III. However, the effect of cyclical mastalgia clearly emerges independently of the personal history of BBD.

### Discussion

In a case-control study concerning 210 cases of newly diagnosed BC cases, and 210 age-, year of birth-, FFTP-, education-matched controls from the same geographic area, four factors were found to be associated with the occurrence of BC, i.e. family history of breast cancer, personal history of BBD, OC use and cyclical mastalgia. Both a family history of BC and a personal history of BBD have already been largely described as BC risk factors (Anderson *et al.*, 1977; Dupont & Page, 1985; Wang & Fentiman, 1985; Kelsey & Berkowitz, 1988; Dupont *et al.*, 1989). The magnitude of the observed effects seems to be in agreement with data reported by these authors.

Initial cohort studies in the seventies and early eighties could not find any increase in BC occurrence in oral contraceptive users (Trapido, 1981; Kay & Hannaford, 1988; Vessey *et al.*, 1989; Romieu *et al.*, 1989). More recently, several authors have brought evidence suggesting a potential association between long term oral contraceptive use before FFTP and later occurrence of BC (Paffenberger *et al.*, 1980; Pike *et al.*, 1981; Meirick *et al.*, 1986; MacPherson *et al.*, 1987*b*; Stadel *et al.*, 1989), although these results have been and are still widely debated (Schlesselman, 1989; Chilvers & Deacon, 1990). Our data are consistent with those findings as we could detect a significant increase of the RR in long term OC users and this tendency was found as well, though attenuated, in long term users before FFTP and, to a lesser extent, in long term users after FFTP. We could not analyse further on the specific effect of LOC and progestin only pill due to insufficient data for exclusive users of those specific OC types. It is noteworthy that the matching design exposed both cases and controls to the same type of OC compounds despite the profound changes in OC composition between the sixties and the eighties.

Cyclical mastalgia as defined in our study was found significantly associated with breast cancer, even after adjustment for the previously mentioned potential confounding factors. This result is rather astonishing and must be interpreted with caution considering the subjectivity of this symptom. The limits of case-control studies have been already largely addressed (Skegg, 1988) and could account entirely for the observed results thus forbiding any generalisation. A possibility of a selection and a recall bias has to be discussed. A recall bias could account for these results: in their attempt to give a rational to the origin of their disease, women diagnosed with BC are thought to be more likely to report breast events than normal women (Skegg *et al.*, 1988). Although we cannot totally dismiss this possibility, our study design and mode of interview tried to minimise it; (1) by chosing as interviewers only qualified female medical doctors; (2) by giving them a specific training in systematically rating breast symptoms. None of the interviewers were aware of the study hypothesis and the interviews of cases and controls were conducted independently. Moreover, our controls were chosen among women enrolled into a chronic health care program on a voluntary basis and were not supposed to be free of breast abnormalities but BC. The absence of the latter disease in this category of urban women, who are aware of the frequency of BC, could have on the opposite encourage them to report minor breast symptoms in order to get a qualified advice about their personal risk of contracting a BC. An increase of the BC RR in relation to the duration of cyclical mastalgia advocates an absence of a strong recall bias effect.

A selection bias can be minimised on the following arguments: first, the restriction of our control group to patients exposed to the same environment, with a matching on year of birth, age at interview, educational level, age at FFTP, when possible, should have limited major environmental factors and birth cohort effect (Lund, 1989); second, as personal history of BBD is known to increase the risk of BC, a higher number of patients with previous history of BBD among the cases could account for the effect of cyclical mastalgia. However, we still observed the effect of cyclical mastalgia after adjustment on BBD and no significant interaction between this factor and BBD could be found.

The statistical analysis was carried out on a 1:1 matched basis thus ensuing equal strata and facilitating analysis. Overmatching was ruled out by our comparison with the global analysis and the simulated study.

Experimental and human data associate breast swelling and pain with exposure to oestrogens. Wisbey *et al.* (1983) noticed that cyclical pain usually begins early in genital life, before the end of the third decade of age and persists for a long time, sometimes resuming at a time of a major hormonal change like pregnancy, but frequently ending only at the onset of the menopause, suggesting its association with individual hormonal secretion characteristics. However it is a clinical evidence in reproductive endocrinology that plasma oestradiol levels do not totally reflect the hormonal impact at the target level. The large inter- and intra-individual variability in the amount of drugs necessary to achieved the same effect at the target level (induction of the LH pulse when monitoring the menstrual cycle; relief of post castration or post menopausal symptoms in oestrogen replacement therapies) could explain the difficulties of studies aiming to demonstrate an excess in oestrogen plasma level, in women with BC (Wysowski *et al.*, 1987; Krieger, 1989). Boyd *et al.* (1988) have shown an association between the fat intake, the cholesterol plasma level, which is a precursor of the sexual hormones, and the severity of the symptom. Other authors have suggested the involvement of prolactin (Watt-Boolson, 1981) or progesterone (Pike *et al.*, 1983), but always pointing out to an hypersensitivity to the oestrogen environment. In our definition cyclical mastalgia is an easy and early marker of breast susceptibility to oestrogen. The present study suggest its predictive value in addition to other classical risk factors of BC. We hope that future cohort studies will include and record accurately this symptom to prove its pertinency.

The authors thank J. Fermanian for its expert assistance in developing the questionnaire, N. Mairon, I. Boucot, M.A. Scardina for conducting the interviews, N. Sterkers, J. Beauvais and M.J. Blin for their expert medical assistance, J. Dalle for allowing us to work in Institut Curie, and B. Falissard for conducting the simulation study.

### APPENDIX

Practical questions asked to the study subjects in order to detect and characterise a cyclical mastalgia. These questions were separately recorded for the immediate period preceding the interview and the other periods of the genital life, as determined by marking events like puberty, first sexual intercouse, first oral contraceptive use, first pregnancy and so on.

- Do (did) you experience breast pain or tenderness?
- Is (was) this breast pain or tenderness bilateral?
- Is (was) the symptom isolated or associated with bilateral breast lumps or nodules?
- Is (was) this breast pain or tenderness associated with an increase in volume of your breast? To what extent? Do (did) you change your bra size? Are (were) you forced to sleep on your back? Does (did) it interfere with your social, professional or private life?
- What is (was) the timing of the symptom: does (did) the symptom disappear or is (was), at least, relieved the day the menstrual bleeding starts? If so, how far from next menses does (did) it start usually? less than 4 days; more than 3 weeks?

– Do (did) you experience this symptom continuously for more than 6 months? When and how it has disappeared?

### References

ANDERSON, D.E. (1977). Breast cancer in families. *Cancer*, **30**, 1855.

BOYD, N.F., SHANNON, P., KRIUKOV, V. & 4 others (1988). Effect of a low-fat high carbohydrate diet on symptoms of cyclical mastopathy. *The Lancet*, **ii**, 128.

BRESLOW, N.E., DAY, N.E., HALVORSEN, K.T., PRENTICE, R.L. & SABAI, C. (1978). Estimation of multiple relative risk functions in matched case control studies. *Am. J. Epidemiol.*, **108**, 299.

BRESLOW, N.E. & DAY, N.E. (1980). The analysis of case control studies. In *IARC Scientific Publication 32*. David, W. (ed.), pp. 162. International Agency for Research on Cancer: Lyon.

BROOKMEYER, R., LIANG, K.Y. & LINET, M. (1986). Matched case-control designs and overmatched analysis. *Am. J. Epidemiol.*, **124**, 693.

CANCER AND STEROID HORMONE STUDY OF THE CENTERS FOR DISEASE CONTROL AND THE NATIONAL INSTITUTE OF CHILD HEALTH AND HUMAN DEVELOPMENT (1986). Oral-contraceptive use and the risk of breast cancer. *N. Engl. J. Med.*, **315**, 405.

CHILVERS, C.E.D. & DEACON, J.M. (1990). Oral contraceptives and breast cancer. *Br. J. Cancer*, **61**, 1.

CLAVEL, F., ANDRIEU, N., GAIRARD, B. & 7 others (1991). Oral contraceptive and breast cancer: a French case-control study. *Int. J. Epidemiol.*, **20**, 32.

COWAN, L.D., GORDIS, L., TONASCIA, J.A. & SEEGARJONES, G. (1981). Breast cancer incidence in women with a history of progesterone deficiency. *Am. J. Epidemiol.*, **114**, 209.

DUPONT, W.D. & PAGE, D.L. (1985). Risk factors for breast cancer in women with proliferative breast disease. *N. Engl. J. Med.*, **312**, 146.

DUPONT, W.D., PAGE, D.L., ROGERS, L.W. & PARL, F.F. (1989). Influence of exogenous estrogens, proliferative breast disease and other variables on breast cancer risk. *Cancer*, **63**, 948.

FENTIMAN, I.S., CALEFFI, M., BRAME, K., CHAUDARY, M.A. & HAYWARD, D.J.L. (1986). Double blind trial of tamoxifen therapy for mastalgia. *Lancet*, **i**, 287.

HENDERSON, B.E., ROSS, R.K., PIKE, M.C. & CASAGRANDE, J.T. (1982). Endogenous hormones as a major factor in human cancer. *Cancer Res.*, **42**, 3232.

KALLNER, G. (1985). An incidence of bilateral mastodynia after the menopause. *Acta Obstet. Gynecol. Scand.*, **64**, 541.

KAY, C.R. & HANNAFORD, P.C. (1988). Breast cancer and the pill – a further report from the Royal College of General Practitioners' oral contraception study. *Br. J. Cancer*, **58**, 675.

KELSEY, J.L. (1979). A review of the epidemiology of human breast cancer. *Epidemiol. Rev.*, **1**, 74.

KELSEY, J.L. & BERKOWITZ, G.S. (1988). Breast cancer epidemiology. *Cancer Res.*, **48**, 5615.

KORENMAN, S. (1980). The endocrinology of breast cancer. *Cancer*, **46**, 874.

KRIEGER, N. (1989). Exposure, susceptibility, and breast cancer risk: a hypothesis regarding exogenous carcinogens, breast tissue development, and social gradients, including black/white differences, in breast cancer incidence. *Breast Cancer Res. & Treat.*, **13**, 205.

LEINSTER, S.J., WHITEHOUSE, G.H. & WALSCH, P.H. (1987). Cyclical mastalgia: clinical and mammographic observations in a screened population. *Br. J. Surg.*, **74**, 220.

LOVE, S.M., GELMAN, R.S. & SILEN, W. (1982). Fibrocystic disease of the breast: a non disease? *N. Engl. J. Med.*, **307**, 1010.

LUND, E. (1989). The validity of different control groups in a case-control study. Oral contraceptive use and breast cancer in young women. *J. Clin. Epidemiol.*, **42**, 987.

MACMAHON, B., COLE, P. & BROWN, J. (1973). Etiology of human breast cancer: a review. *J. Natl Cancer Inst.*, **50**, 21.

MACPHERSON, K., COOPE, P.A. & VESSEY, M.P. (1987*a*). Early oral contraceptive use and breast cancer: theorical effects of latency. *J. Epidem. Community Health*, **36**, 595.

MACPHERSON, K., VESSEY, M.P., NEIL, A., DOLL, R., JONES, L. & ROBERTS, M. (1987*b*). Early oral contraceptive use and breast cancer: results of another case-control study. *Br. J. Cancer*, **56**, 653.

MADDOX, P.R. (1989). The management of mastalgia in U.K. *Horm. Metab. Res.*, **32** (Suppl), 21.

MEIRIK, O., LUND, E., ADAMI, H.O., BERGSTROM, R., CHRISTOFFERSEN, T. & BERGSJO, P. (1986). Oral contraceptive and breast cancer in young women: a joint national case-control study in Sweden and Norway. *Lancet*, **ii**, 650.

MILLER, D.R., ROSENBERG, L., KAUFFMAN, D.W., STOLLEY, P., WARSHAUEER, M.E. & SHAPIRO, S. (1989). Breast cancer before age 45 and oral contraceptive use: new findings. *Am. J. Epidemiol.*, **129**, 269.

MUIR, C.S. (1990). Epidemiology, basic science and the prevention of cancer: implications for the future. *Cancer Res.*, **50**, 6441.

OLSSON, H., MOLLER, T.R. & RANSTAM, J. (1989). Early oral contraceptive use and breast cancer among premenopausal women: final report from a study in southern Sweden. *J. Natl Cancer Inst.*, **81**, 1000.

PAFFENBARGER, R.S., KAMPERT, J.B. & CHANG, H.G. (1980). Characteristics that predict risk of breast cancer before and after the menopause. *Am. J. Epidemiol.*, **112**, 258.

PIKE, M.C., HENDERSON, B.E., CASAGRANDE, J.T., ROSARIO, I. & RAY, G.E. (1981). Oral contraceptive use and early abortion as risk factors for breast cancer in young women. *Br. J. Cancer*, **43**, 72.

PIKE, M.C., HENDERSON, B.E., KRAILO, M.D., DUKE, A. & ROY, S. (1983). Breast cancer in young women and use in oral contraception: possible modifying effect of formulation and age at use. *Lancet*, **ii**, 926.

PREECE, P.E., HUGHES, L.E. & MANSEL, R.E. (1976). Clinical syndromes of mastalgia. *Lancet*, **ii**, 670.

PREECE, P.E., MANSEL, R.E., HUGHES, L.E., BAUM, M., BOLTON, P.M. & GRAVELLE, I.H. (1978). Mastalgia: psychoneurosis or organic disease? *Br. Med. J.*, **1**, 29.

ROMIEU, I., WILLETT, W.C. & COLDITZ, G.A. & 4 others (1989). Prospective study of oral contraceptive use and risk of breast cancer in women. *J. Natl Cancer Inst.*, **81**, 1313.

SCHLESSELMAN, J.J. (1989). Cancer of the breast and reproductive tract in relation to use of oral contraceptives. *Contraception*, **40**, 1.

SITRUK-WARE, R., STERKERS, N., MOWSZOWICZ, I. & MAUVAIS-JARVIS, P. (1977). Inadequate corpus luteal function in women with benign breast disease. *J. Clin. Endocrinol. Metab.*, **44**, 771.

SKEGG, D.C.G. (1988). Potential for bias in case-control studies of oral contraceptives and breast cancer. *Am. J. Epidemiol.*, **127**, 205.

STADEL, B.V., SCHLESSELMAN, J.J. & MURRAY, P.A. (1989). Oral contraceptives and breast cancer. *Lancet*, **i**, 1257.

THALABARD, J.C., PLU-BUREAU, G. & FALISSARD, B. (1989). A program running on MS-DOS computer for the analysis of epidemiologic stratified data. *Comp. Meth. Prog. Biomed.*, **28**, 191.

TRAPIDO, E.J. (1981). A prospective study of oral contraceptives and breast cancer. *J. Natl Cancer Inst.*, **67**, 1011.

UK NATIONAL CASE-CONTROL STUDY GROUP (1989). Oral contraceptive use and breast cancer risk in young women. *Lancet*, **i**, 973.

VESSEY, M.P., MACPHERSON, K., VILLARD-MACKINTOSH, L. & YEATES, D. (1989). Oral contraceptives and breast cancer: latest findings in a large cohort study. *Br. J. Cancer*, **59**, 613.

VORHERR, H. (1986). Fibrocystic breast disease: pathophysiology, pathomorphology, clinical picture and management. *Am. J. Obstet. Gynecol.*, **154**, 161.

WANG, D.Y. & FENTIMAN, I.S. (1985). Epidemiology and endocrinology of benign breast disease. *Breast Cancer Res. & Treat.*, **6**, 5.

WATT-BOOLSEN, S., ANDERSON, A.N. & BLICHERT-TOFT, M. (1981). Serum prolactin and oestradiol levels in women with cyclical mastalgia. *Horm. Metab. Res.*, **13**, 700.

WISBEY, J.R., MANSEL, R.E., PYE, J.K., KUMAR, S., PREECE, P.E. & HUGHES, L.E. (1983). Natural history of breast pain. *Lancet*, **ii**, 672.

WYSOWSKI, D.K., COMSTOCK, G.W., HELSING, K.J. & LAU, H.L. (1987). Sex hormone levels in serum in relation to the development of breast cancer. *Am. J. Epidemiol.*, **125**, 791.


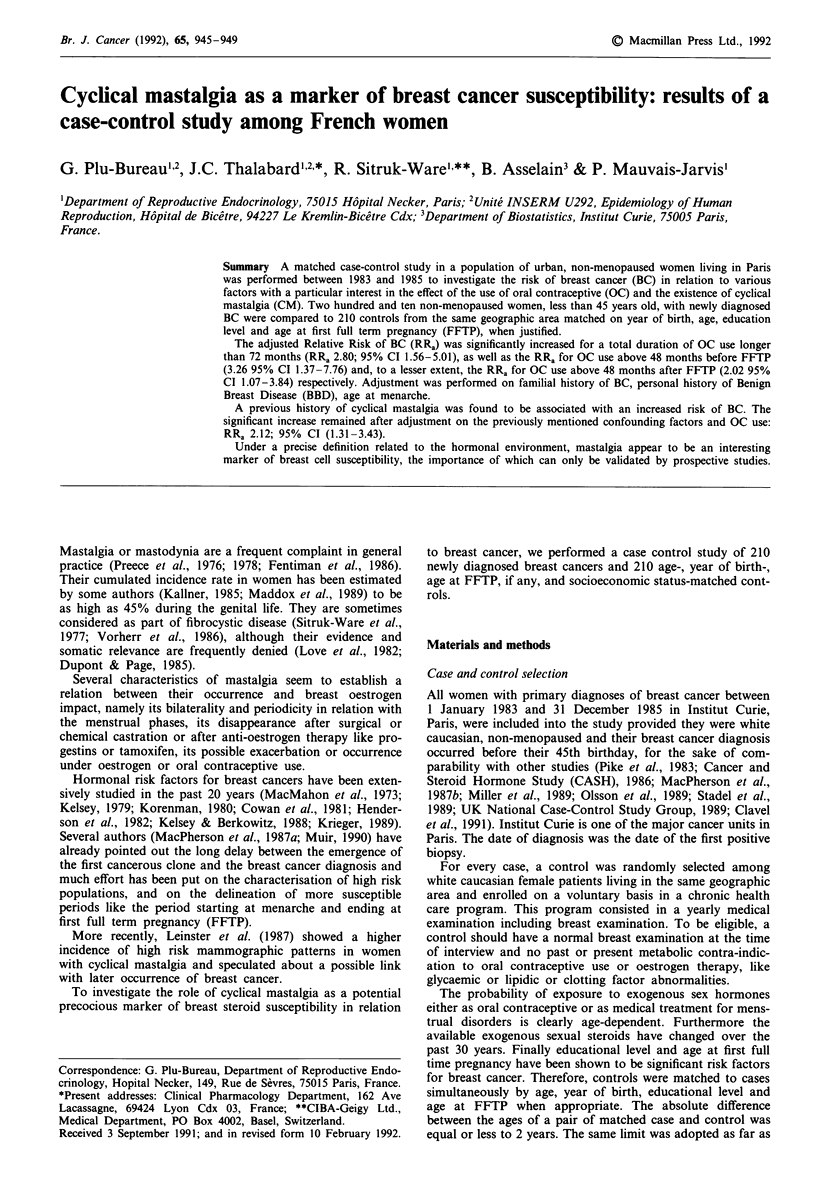

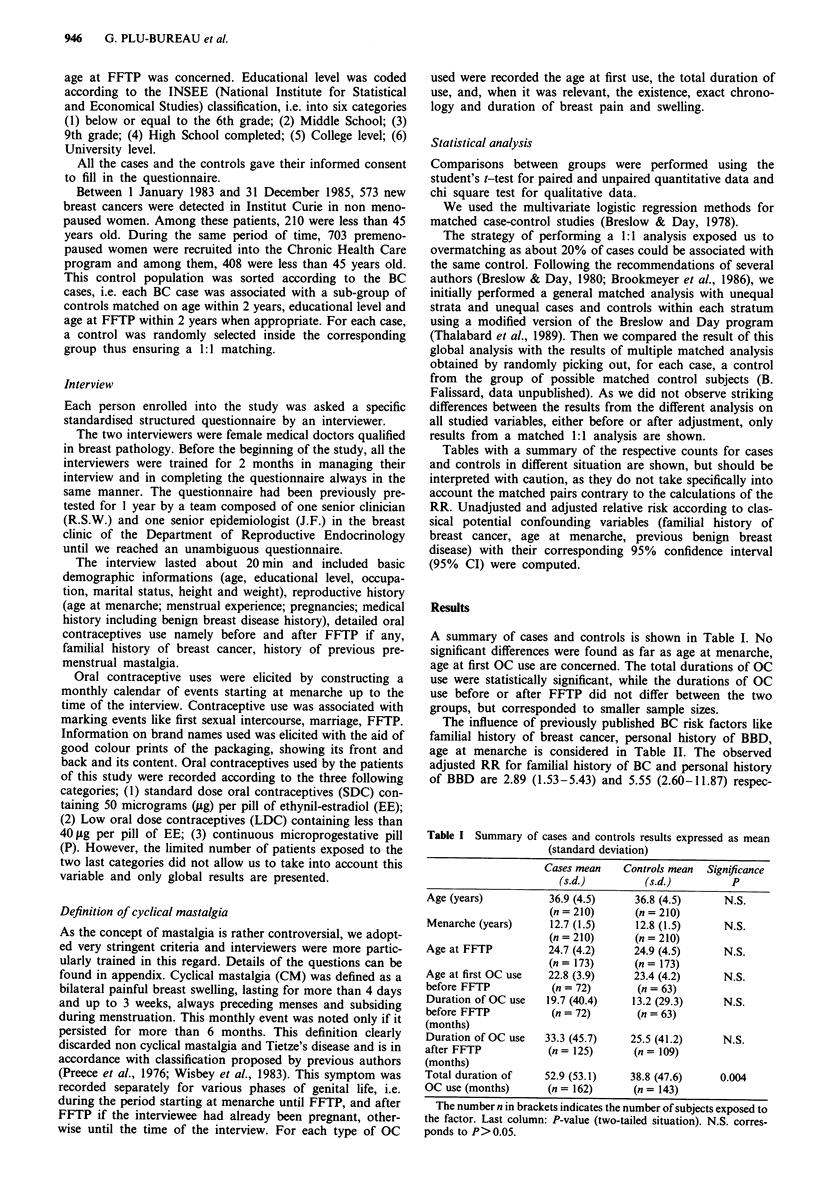

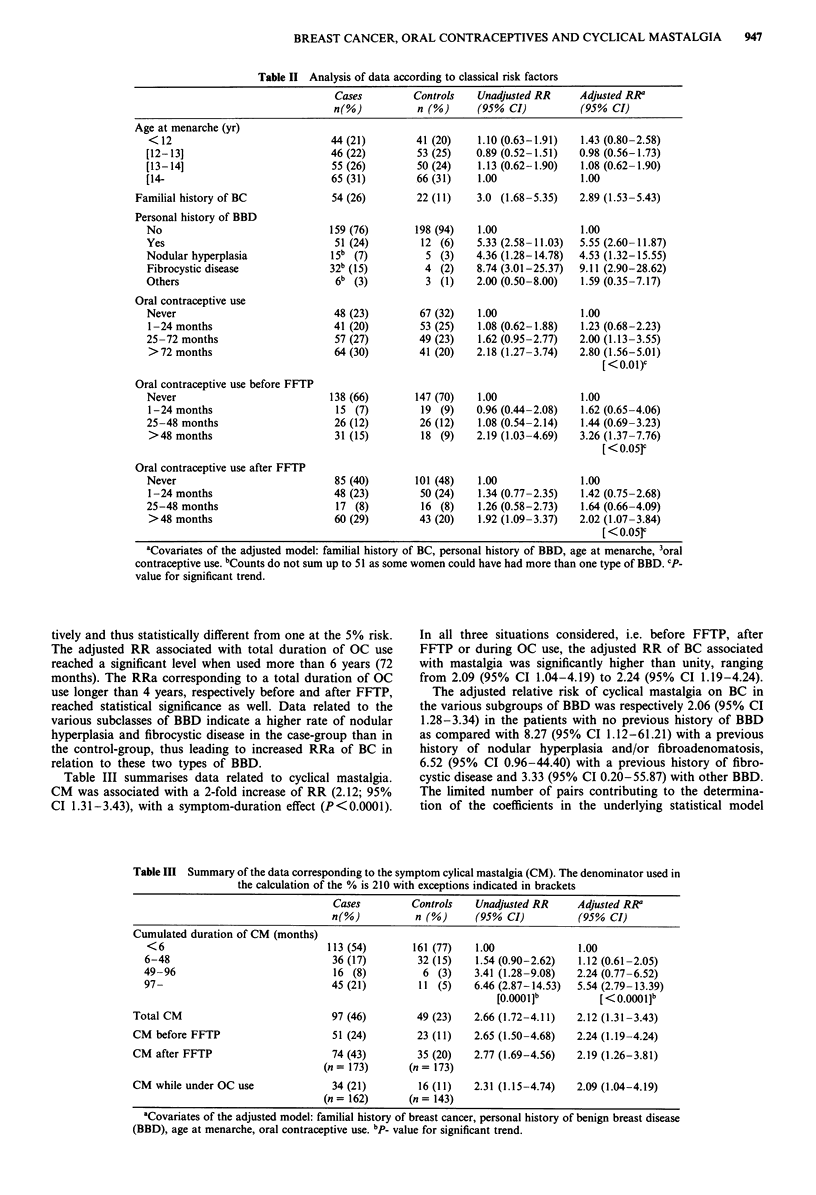

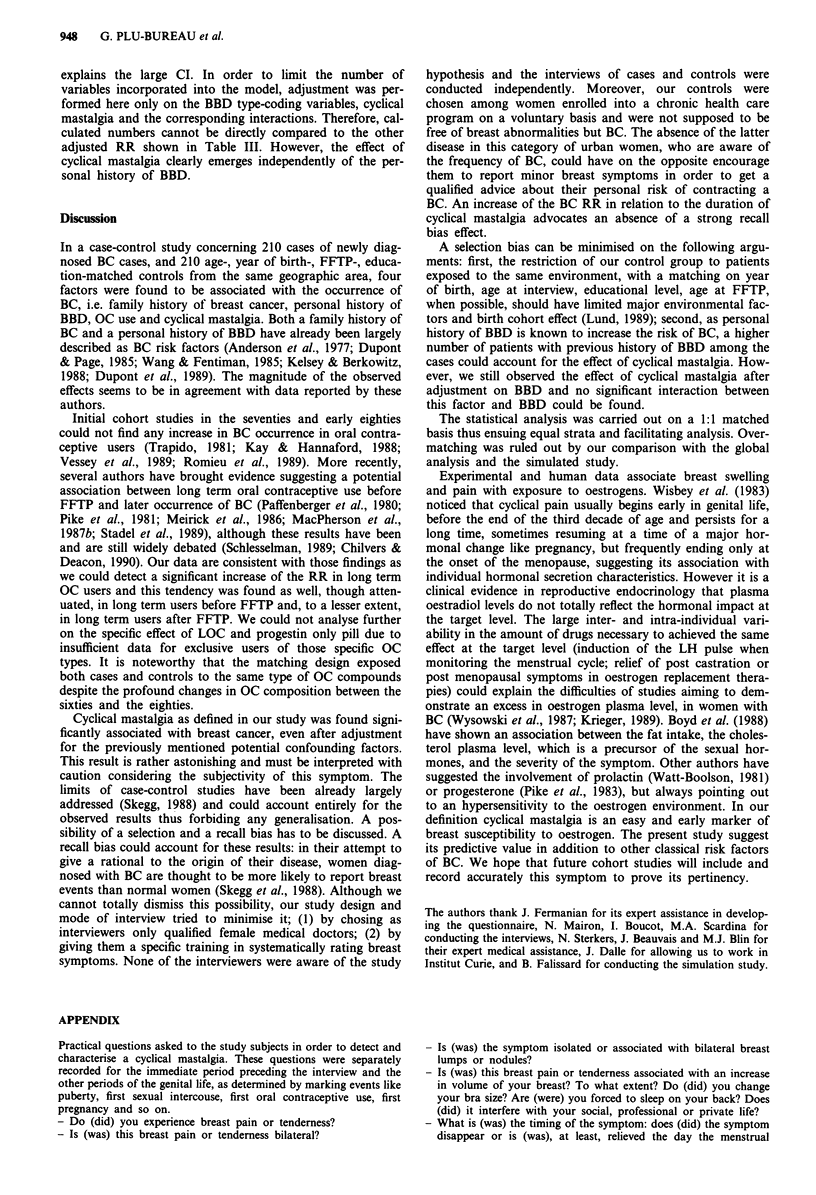

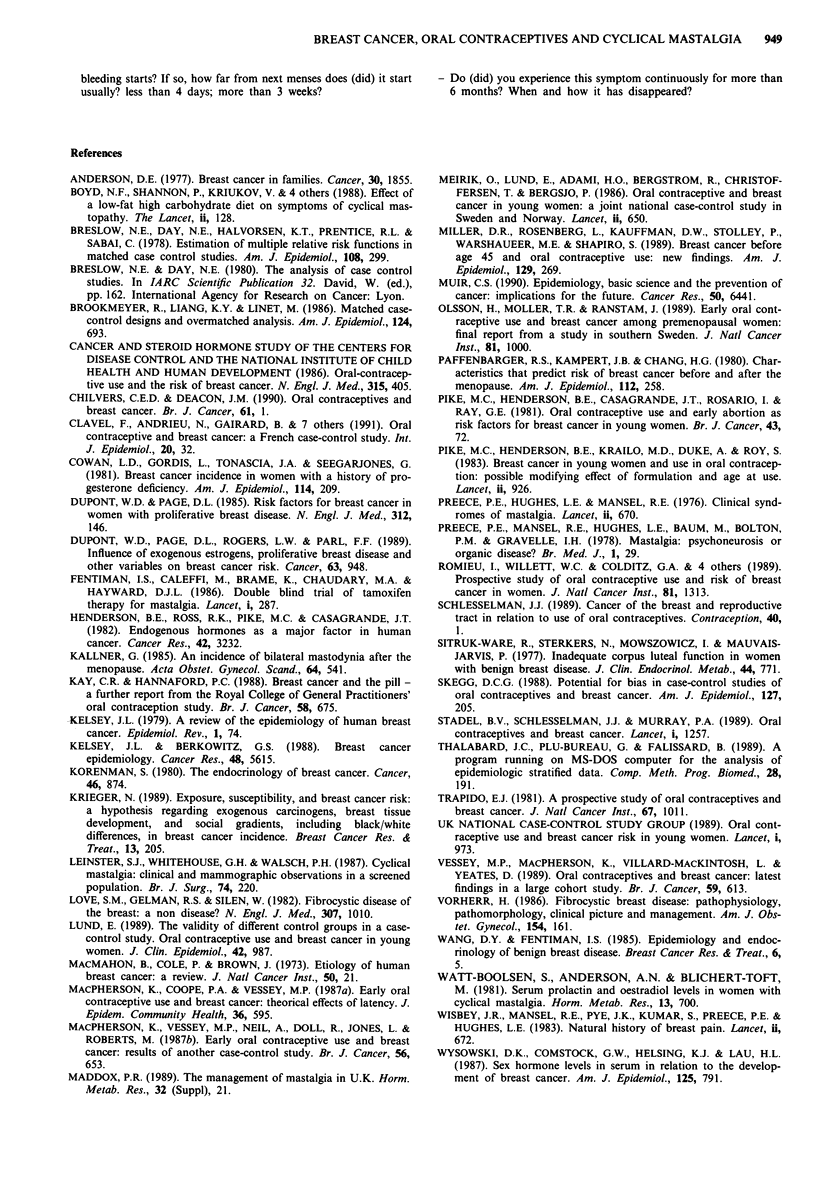


## References

[OCR_00587] Anderson D. E. (1977). Breast cancer in families.. Cancer.

[OCR_00588] Boyd N. F., McGuire V., Shannon P., Cousins M., Kriukov V., Mahoney L., Fish E., Lickley L., Lockwood G., Tritchler D. (1988). Effect of a low-fat high-carbohydrate diet on symptoms of cyclical mastopathy.. Lancet.

[OCR_00593] Breslow N. E., Day N. E., Halvorsen K. T., Prentice R. L., Sabai C. (1978). Estimation of multiple relative risk functions in matched case-control studies.. Am J Epidemiol.

[OCR_00603] Brookmeyer R., Liang K. Y., Linet M. (1986). Matched case-control designs and overmatched analyses.. Am J Epidemiol.

[OCR_00613] Chilvers C. E., Deacon J. M. (1990). Oral contraceptives and breast cancer.. Br J Cancer.

[OCR_00622] Cowan L. D., Gordis L., Tonascia J. A., Jones G. S. (1981). Breast cancer incidence in women with a history of progesterone deficiency.. Am J Epidemiol.

[OCR_00627] Dupont W. D., Page D. L. (1985). Risk factors for breast cancer in women with proliferative breast disease.. N Engl J Med.

[OCR_00632] Dupont W. D., Page D. L., Rogers L. W., Parl F. F. (1989). Influence of exogenous estrogens, proliferative breast disease, and other variables on breast cancer risk.. Cancer.

[OCR_00637] Fentiman I. S., Caleffi M., Brame K., Chaudary M. A., Hayward J. L. (1986). Double-blind controlled trial of tamoxifen therapy for mastalgia.. Lancet.

[OCR_00642] Henderson B. E., Ross R. K., Pike M. C., Casagrande J. T. (1982). Endogenous hormones as a major factor in human cancer.. Cancer Res.

[OCR_00647] Kallner G. (1985). An incident of bilateral mastodynia after the menopause.. Acta Obstet Gynecol Scand.

[OCR_00651] Kay C. R., Hannaford P. C. (1988). Breast cancer and the pill--a further report from the Royal College of General Practitioners' oral contraception study.. Br J Cancer.

[OCR_00656] Kelsey J. L. (1979). A review of the epidemiology of human breast cancer.. Epidemiol Rev.

[OCR_00660] Kelsey J. L., Berkowitz G. S. (1988). Breast cancer epidemiology.. Cancer Res.

[OCR_00664] Korenman S. G. (1980). The endocrinology of breast cancer.. Cancer.

[OCR_00668] Krieger N. (1989). Exposure, susceptibility, and breast cancer risk: a hypothesis regarding exogenous carcinogens, breast tissue development, and social gradients, including black/white differences, in breast cancer incidence.. Breast Cancer Res Treat.

[OCR_00675] Leinster S. J., Whitehouse G. H., Walsh P. V. (1987). Cyclical mastalgia: clinical and mammographic observations in a screened population.. Br J Surg.

[OCR_00680] Love S. M., Gelman R. S., Silen W. (1982). Sounding board. Fibrocystic "disease" of the breast--a nondisease?. N Engl J Med.

[OCR_00684] Lund E. (1989). The validity of different control groups in a case-control study. Oral contraceptive use and breast cancer in young women.. J Clin Epidemiol.

[OCR_00689] MacMahon B., Cole P., Brown J. (1973). Etiology of human breast cancer: a review.. J Natl Cancer Inst.

[OCR_00704] Maddox P. R. (1989). The management of mastalgia in the UK.. Horm Res.

[OCR_00698] McPherson K., Vessey M. P., Neil A., Doll R., Jones L., Roberts M. (1987). Early oral contraceptive use and breast cancer: results of another case-control study.. Br J Cancer.

[OCR_00710] Meirik O., Lund E., Adami H. O., Bergström R., Christoffersen T., Bergsjö P. (1986). Oral contraceptive use and breast cancer in young women. A joint national case-control study in Sweden and Norway.. Lancet.

[OCR_00714] Miller D. R., Rosenberg L., Kaufman D. W., Stolley P., Warshauer M. E., Shapiro S. (1989). Breast cancer before age 45 and oral contraceptive use: new findings.. Am J Epidemiol.

[OCR_00720] Muir C. S. (1990). Epidemiology, basic science, and the prevention of cancer: implications for the future.. Cancer Res.

[OCR_00724] Olsson H., Möller T. R., Ranstam J. (1989). Early oral contraceptive use and breast cancer among premenopausal women: final report from a study in southern Sweden.. J Natl Cancer Inst.

[OCR_00775] (1989). Oral contraceptives and breast cancer.. Lancet.

[OCR_00730] Paffenbarger R. S., Kampert J. B., Chang H. G. (1980). Characteristics that predict risk of breast cancer before and after the menopause.. Am J Epidemiol.

[OCR_00735] Pike M. C., Henderson B. E., Casagrande J. T., Rosario I., Gray G. E. (1981). Oral contraceptive use and early abortion as risk factors for breast cancer in young women.. Br J Cancer.

[OCR_00741] Pike M. C., Henderson B. E., Krailo M. D., Duke A., Roy S. (1983). Breast cancer in young women and use of oral contraceptives: possible modifying effect of formulation and age at use.. Lancet.

[OCR_00747] Preece P. E., Mansel R. E., Bolton P. M., Hughes L. M., Baum M., Gravelle I. H. (1976). Clinical syndromes of mastalgia.. Lancet.

[OCR_00751] Preece P. E., Mansel R. E., Hughes L. E. (1978). Mastalgia: psychoneurosis or organic disease?. Br Med J.

[OCR_00756] Romieu I., Willett W. C., Colditz G. A., Stampfer M. J., Rosner B., Hennekens C. H., Speizer F. E. (1989). Prospective study of oral contraceptive use and risk of breast cancer in women.. J Natl Cancer Inst.

[OCR_00761] Schlesselman J. J. (1989). Cancer of the breast and reproductive tract in relation to use of oral contraceptives.. Contraception.

[OCR_00768] Sitruk-ware L. R., Sterkers N., Mowszowicz I., Mauvais-Jarvis P. (1977). Inadequate corpus luteal function in women with benign breast diseases.. J Clin Endocrinol Metab.

[OCR_00770] Skegg D. C. (1988). Potential for bias in case-control studies of oral contraceptives and breast cancer.. Am J Epidemiol.

[OCR_00779] Thalabard J. C., Plu-Bureau G., Falissard B. (1989). A program running under MS-DOS for the analysis of epidemiologic stratified or matched data.. Comput Methods Programs Biomed.

[OCR_00785] Trapido E. J. (1981). A prospective cohort study of oral contraceptives and breast cancer.. J Natl Cancer Inst.

[OCR_00794] Vessey M. P., McPherson K., Villard-Mackintosh L., Yeates D. (1989). Oral contraceptives and breast cancer: latest findings in a large cohort study.. Br J Cancer.

[OCR_00799] Vorherr H. (1986). Fibrocystic breast disease: pathophysiology, pathomorphology, clinical picture, and management.. Am J Obstet Gynecol.

[OCR_00804] Wang D. Y., Fentiman I. S. (1985). Epidemiology and endocrinology of benign breast disease.. Breast Cancer Res Treat.

[OCR_00809] Watt-Boolsen S., Andersen A. N., Blichert-Toft M. (1981). Serum prolactin and oestradiol levels in women with cyclical mastalgia.. Horm Metab Res.

[OCR_00814] Wisbey J. R., Kumar S., Mansel R. E., Peece P. E., Pye J. K., Hughes L. E. (1983). Natural history of breast pain.. Lancet.

[OCR_00819] Wysowski D. K., Comstock G. W., Helsing K. J., Lau H. L. (1987). Sex hormone levels in serum in relation to the development of breast cancer.. Am J Epidemiol.

